# Deriving alpha angle from anterior-posterior dual-energy x-ray absorptiometry scans: an automated and validated approach

**DOI:** 10.12688/wellcomeopenres.16656.2

**Published:** 2022-07-19

**Authors:** Benjamin G. Faber, Raja Ebsim, Fiona R. Saunders, Monika Frysz, George Davey Smith, Timothy Cootes, Jonathan H. Tobias, Claudia Lindner

**Affiliations:** 1 Musculoskeletal Research Unit, University of Bristol, Bristol, UK; 2 Medical Research Council Integrative Epidemiology Unit, University of Bristol, Bristol, UK; 3 Division of Informatics, Imaging and Data Sciences, University of Manchester, Manchester, UK; 4 Centre for Arthritis and Musculoskeletal Health, University of Aberdeen, Aberdeen, UK

**Keywords:** Alpha Angle, DXA, Automation, Cam, Femoro-acetabular impingement

## Abstract

**Introduction:** Alpha angle (AA) is a widely used imaging measure of hip shape that is commonly used to define cam morphology, a bulging of the lateral aspect of the femoral head. Cam morphology has shown strong associations with hip osteoarthritis (OA) making the AA a clinically relevant measure. In both clinical practice and research studies, AA tends to be measured manually which can be inconsistent and time-consuming.

**Objective:** We aimed to (i) develop an automated method of deriving AA from anterior-posterior dual-energy x-ray absorptiometry (DXA) scans; and (ii) validate this method against manual measures of AA.

**Methods:** 6,807 individuals with left hip DXAs were selected from UK Biobank. Outline points were manually placed around the femoral head on 1,930 images before training a Random Forest-based algorithm to place the points on a further 4,877 images. An automatic method for calculating AA was written in Python 3 utilising these outline points. An iterative approach was taken to developing and validating the method, testing the automated measures against independent batches of manually measured images in sequential experiments.

**Results:** Over the course of six experimental stages the concordance correlation coefficient, when comparing the automatic AA to manual measures of AA, improved from 0.28 [95% confidence interval 0.13-0.43] for the initial version to 0.88 [0.84-0.92] for the final version. The inter-rater kappa statistic comparing automatic versus manual measures of cam morphology, defined as AA ³≥60°, improved from 0.43 [80% agreement] for the initial version to 0.86 [94% agreement] for the final version.

**Conclusions:** We have developed and validated an automated measure of AA from DXA scans, showing high agreement with manually measuring AA. The proposed method is available to the wider research community from Zenodo.

## Introduction

**Figure 1.**
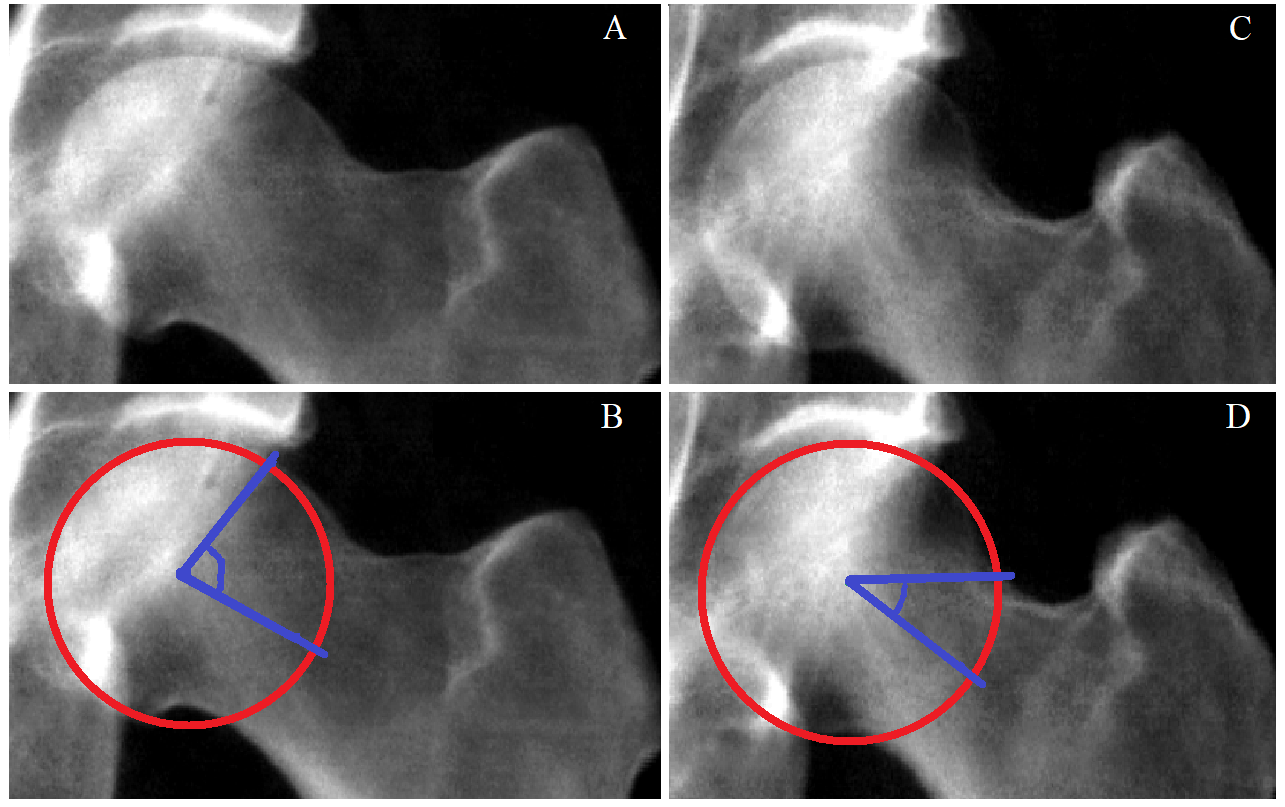
Alpha angle depicted on UK Biobank hip dual-energy absorptiometry scans. **A**) A representative UK Biobank (UKB) hip dual-energy x-ray absorptiometry (DXA) scan depicting a femoral head with cam morphology. **B**) The same DXA as in A marked with a red circle of best fit plotted over the femoral head. The two blue lines illustrate the lines from which the alpha angle (AA) is calculated; one line goes from the middle of the femoral neck to the centre of the circle and the other goes from the centre of the circle to the point at which the femoral head leaves the circle of best fit. **C**) A UKB hip DXA without cam morphology. **D**) The same DXA as in C marked with a red circle of best fit and blue lines from which the AA is calculated. Bottom left image: The same DXA as above marked with a red circle of best fit plotted over the femoral head. The two blue lines illustrate the lines from which the alpha angle (AA) is calculated; one line goes from the middle of the femoral neck to the centre of the circle and the other goes from the centre of the circle to the point at which the femoral head leaves the circle of best fit. Top right image: A UKB hip DXA without cam morphology. Bottom right image: The same DXA as above marked with a red circle of best fit and blue lines from which the AA is calculated.

Alpha angle (AA) is a measure designed to examine the presence and severity of cam morphology at the hip joint ^1^. Cam morphology describes a bulging of the lateral aspect of the femoral head that causes the femoral head to become aspherical leading to a pistol grip type appearance; it is a key component of femoro-acetabular impingement (FAI) ^2, 3^. AA is the angle measured between two lines, the first line from the mid-point of the femoral neck to the centre of the femoral head, and a second line from the centre of the femoral head to a point on the femoral head where the femoral head or neck leaves a circle of best fit placed over the femoral head ( Figure 1) ^1, 4^. The higher the AA the more indicative of cam morphology it is – with previously published thresholds of 50°, 55°, 60° and 83° all being used to define the presence of cam morphology ^1, 4– 7^, and with a recent systematic review concluding a threshold of 60° is best ^8^.

Cam morphology is an important shape variation of the femoral head. It has been associated with hip osteoarthritis (OA) and subsequent total hip replacement (THR), a proxy for end-stage OA ^4, 9^. Cam morphology is thought to lead to pain and limited function of the hip in FAI syndrome ^10^. AA has been used in clinical trials as inclusion criteria to investigate the use of surgical procedures to treat FAI syndrome, but no agreed standardised approach of measuring AA exists ^7, 11^. AA was first developed utilising magnetic resonance imaging (MRI) before being applied to anterior-posterior (AP) and lateral radiographs for large-scale epidemiological analyses ^1, 6^. One approach to manually measuring AA on AP hip radiographs is to use software such as HipMorf or OxMorf ^12– 14^. These packages allow the user to manually fit a circle over the femoral head and then mark where the femoral head leaves this. Alternatively, AA has been calculated using MATLAB based software which utilises outline points that have been placed manually around the femoral head ^4, 9^. AA derived from outline points has been shown to be predictive of hip OA ^9^. When using automatically placed points, concerns have been raised about the validity and reproducibility of such an outline points-based approach due to the difficulty in deciding where exactly the femoral head deviates from the circle of best fit ^15^. To date, no reproducibility studies on methods for automatically measuring AA have been published nor is any open source code to do this available.

Dual-energy x-ray absorptiometry (DXA) scans are commonly used to derive measures of bone mineral density, and are increasingly being used to look at hip shape ^16, 17^. In addition, a new generation of hip DXA scanner allows for higher resolution images which has led to the use of DXA scans in detecting radiographic OA for research purposes ^18, 19^. Compared to radiographs, DXA scans involve lower radiation doses and are available from larger population studies, such as the UK Biobank (UKB) enhanced imaging study ^20, 21^. This work aimed to (i) develop a method to automatically derive AA from outline points placed around the femoral head in DXA scans obtained in UKB; and (ii) validate the method against manual measures of AA. We also compare values obtained using our method to previously published population level statistics.

## Methods

### Population

UKB is a UK-based mixed sex cohort made up of 500,000 individuals aged between 40–69 years at recruitment (2006–2010) ^22^. A full data catalogue is available online. A subset of 100,000 individuals are due to have high resolution iDXA scans done on both hips (2013 – ongoing) with over 45,000 already completed ^20^. An initial training sample of 2,000 individuals with a DXA scan was selected but 70 DXA scans were excluded due to poor image quality leaving a training sample of 1,930 individuals. A further extension sample of 5,000 individuals with a DXA scan was selected but 123 DXA scans were excluded due to poor image quality leaving an extension sample of 4,877 individuals. The training and extension samples were selected from an overall sample of 13,496 individuals with DXA scans available at the time (January 2019). The first 20% of both the training and extension samples were selected randomly from those with a self-reported diagnosis of OA based on a questionnaire completed at the same visit as the DXA scan. In the questionnaire, each participant was asked to record all their known diagnoses and a trained nurse subsequently coded the diagnosis. The OA code was not site specific. This was done to increase the number of pathological scans in the training sample as part of a wider research programme to automate the assessment of radiographic osteoarthritis. It was this wider research programme that guided the sample size selection as large samples are needed for machine learning. The remainder of the training and extension samples (80%) were selected randomly ensuring the sexes were equally weighted ^19^. The combined sample is made up of both the training and extension sample. All demographic information was obtained on the same day as the DXA scan. This study was approved by UKB (application number 17295) which is overseen by its own Ethics Advisory Committee. In addition, UKB received approval from the National Information Governance Board for Health and Social Care and Northwest Multi-Centre Research Ethics Committee (11/NW/0382). All participants provided informed consent.

### DXA images and outline points

**Figure 2.**
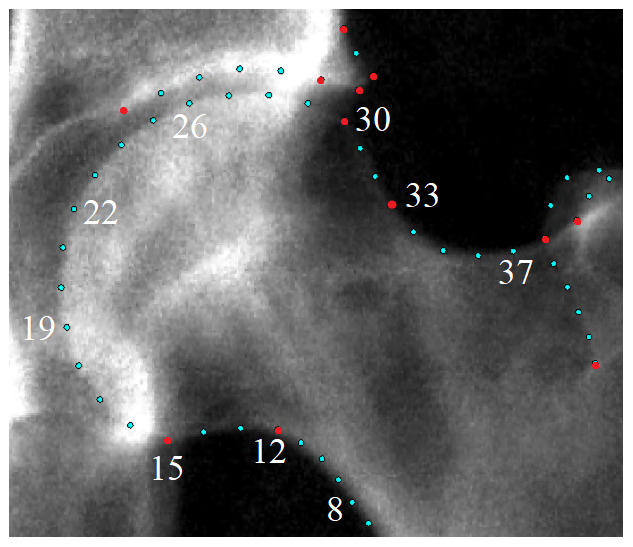
A representative dual-energy x-ray absorptiometry (DXA) scan from UK Biobank (UKB) with all femoral head and acetabular outline points. The points marked in red are key anatomical landmarks. Only points 8-39 were used in this study. The acetabular points and the remaining proximal femur points were not necessary.

As part of UKB, DXA scans of both hips (iDXA GE-Lunar, Madison, WI) were obtained from participants positioned supine with 15–25° internal rotation using a standardised protocol ^21^. In this study, we only examined the left hip DXA scans. All DXAs in the training sample had 85 outline points positioned around the femoral head, metaphysis, lesser and greater trochanters, and the superior acetabulum by four manual annotators. Of the 85 points, 18 points were placed on anatomical landmarks (key points) and the remaining points were placed equidistant apart along the edge of the bone. A Random Forest-based machine learning algorithm was then trained on these images and used to automatically annotate the extension sample with the 85 outline points ^23, 24^. All automatically placed points were checked and manually corrected where necessary. The mean correction distance was 0.7mm (movement orthogonal to bone boundary: 0.1mm) with the majority of points remaining unchanged. When osteophytes were present the outline points were moved manually inside of the osteophyte (if not already correct) to avoid including osteophytes in our AA. Of the 85 outline points only points 8 to 39 along the femoral head and neck were used in this study to derive the AA measurements ( Figure 2). For each image, all point positions were stored as x, y coordinates in a text file.

### Manual measure of alpha angle

**Figure 3.**
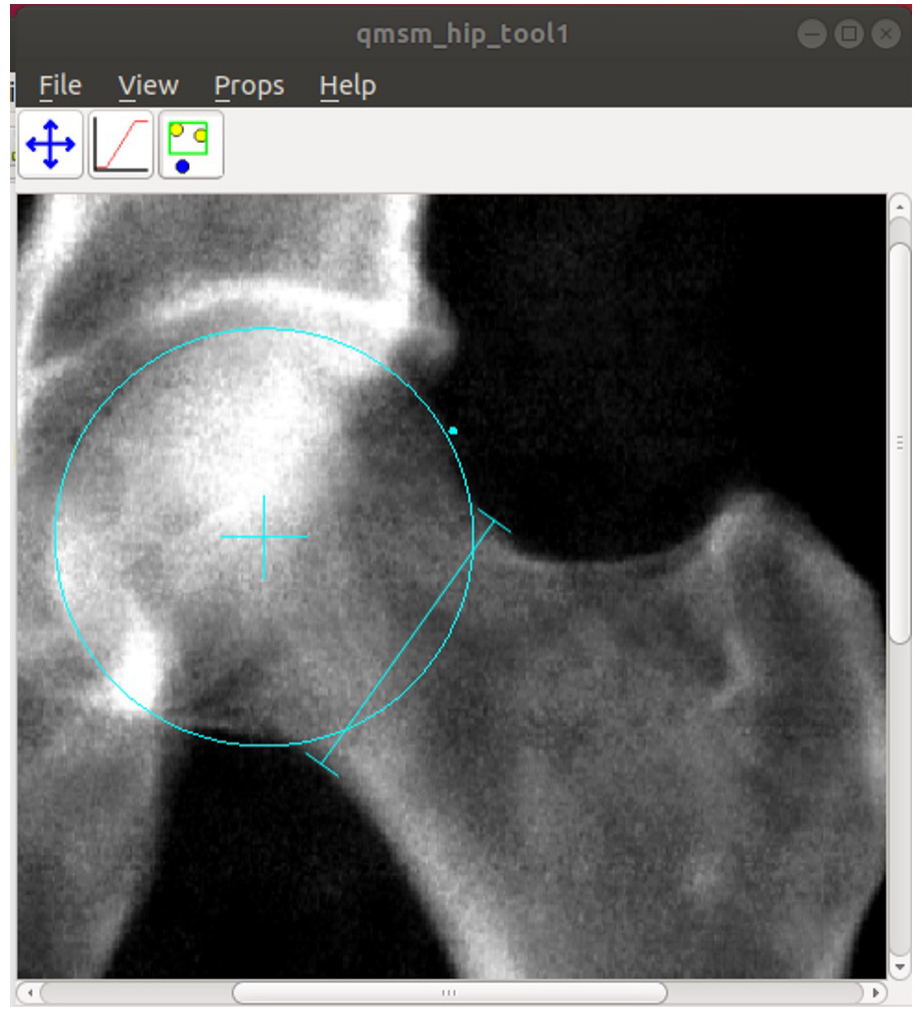
A screenshot of customised software from the University of Manchester. A circle is manually scaled and moved to fit the circle of best fit to the femoral head. A point is placed where the femoral head leaves the circle (cyan dot). Callipers are positioned across the narrowest section of the femoral neck. The software saves the centre point of the circle (cyan cross), the narrowest point of the femoral neck, and the point at which the femoral head deviates from the circle of best fit. The manual AA is then calculated from these points.

To provide a manual ‘gold-standard,’ against which to test the automated method, AA was measured manually by BF, a rheumatology doctor, for a random selection of images divided into 5 batches of 100 (n=400 from the training sample and n=100 from the expansion sample). This was done using custom software (University of Manchester) that allows the user to manually (i) place and scale a circle to best fit the femoral head; (ii) place a point where the femoral head leaves the circle; and (iii) position callipers across the narrowest section of the femoral neck ( Figure 3). The software saves the centre point of the circle, the midpoint of the narrowest section of the femoral neck, and the point at which the femoral head leaves the circle. The manual AA was then calculated from these points using a custom Python 3 script. Intra-rater variability was assessed on a subset of 100 scans, repeating the measurements 9 months after they were initially obtained.

### Automated measure of alpha angle - model refinement approach

We followed a sequential experimental design to test and iteratively optimise our automatic AA calculation based on comparisons with manually derived measures. Initially, a model was designed to derive automatic AA ( *model 1)* and was tested on 100 images randomly selected from the training sample with manually placed outline points. Refinements were made to the way AA was automatically measured *(models 2–4)* and are detailed in the following methods *.* To test each model iteration a subset of 100 images was analysed, with AA measured both manually and automatically in a blinded fashion. The method to automatically measure AA was finalised using subsets of the training sample with manually placed outline points resulting in *model 4*. Following this, a final evaluation was done to analyse the performance of the method when using automatically placed but manually corrected outline points using the extension sample.

### Model 1 - defining a circle of best fit

All point position text files were read into Python 3 using Jupyter Notebook. Within Python 3, a freely available least-squares regression model package ( *circle_fit*) was used to place a circle of best fit to points 15 and 28 on the femoral head ( Figure 2) ^25^. The *circle_fit* output gives the *x and y coordinates of the centre of the circle, the radius of the circle and the variance (the variance was not used).* Point 15 marked the inferior medial curvature of the femoral head and was chosen to be the starting point of the circle fitting. Point 30 marked the superior lateral curvature of the femoral head. However, instead of point 30, point 28 (i.e. two points medial of point 30) was chosen to be the end point of the circle fitting to avoid overfitting the circle of best fit to cam-type femoral heads. This method of circle fitting was manually qualitatively assessed on 500 DXA scans and deemed appropriate.

### Model 1 - defining the femoral neck mid-point

Finding the narrowest point of the femoral neck was done using a line-segment approach. The femoral neck was demarcated by points 8–12 for the medial side and points 32–36 on the lateral side ( Figure 2). For these two sets of points, a straight line was constructed between each pair of consecutive points. For each straight line segment, the shortest distance was measured between that line and a point on the opposing side of the femoral neck. For example, a line would be drawn between points 8 & 9 and the shortest distance may be found between this line and point 35. The shortest distance across all line segments defined the narrowest width of the femoral neck; the mid-point on this line is calculated and saved as the femoral neck point.

### Model 1 - defining the index point

The index point is referred to as the first outline point judged to be truly ‘outside’ of the circle of best fit. It is critical to defining the intersection position, the coordinates at which the femoral head or neck leaves the circle which is the key element for calculating AA. For identifying the index point, we defined the residual as the distance between each point (from points 15–28) and the centre of the circle (distance to centre) minus the radius of the circle. For each image, the maximum residual is the index point threshold for that image. For spherical femoral heads the index point threshold will be lower compared to aspherical femoral heads which are harder to fit a circle to. The index point was defined to be the first point after point 28 which deviated from the circle by more than the index point threshold (i.e. the maximum residual) with the point afterwards (in clockwise direction) also having a residual greater than that of the index point. For example, if point 30 deviated from the circle by more than the maximum residual and point 31 also deviated from the circle by more than point 30’s residual then point 30 would be the index point. In contrast, if the residual of point 31 was smaller than or equal to that of point 30 then point 30 would not be considered to have left the circle and therefore not be the index point.

### Model 1 - defining the intersection position

**Figure 4.**
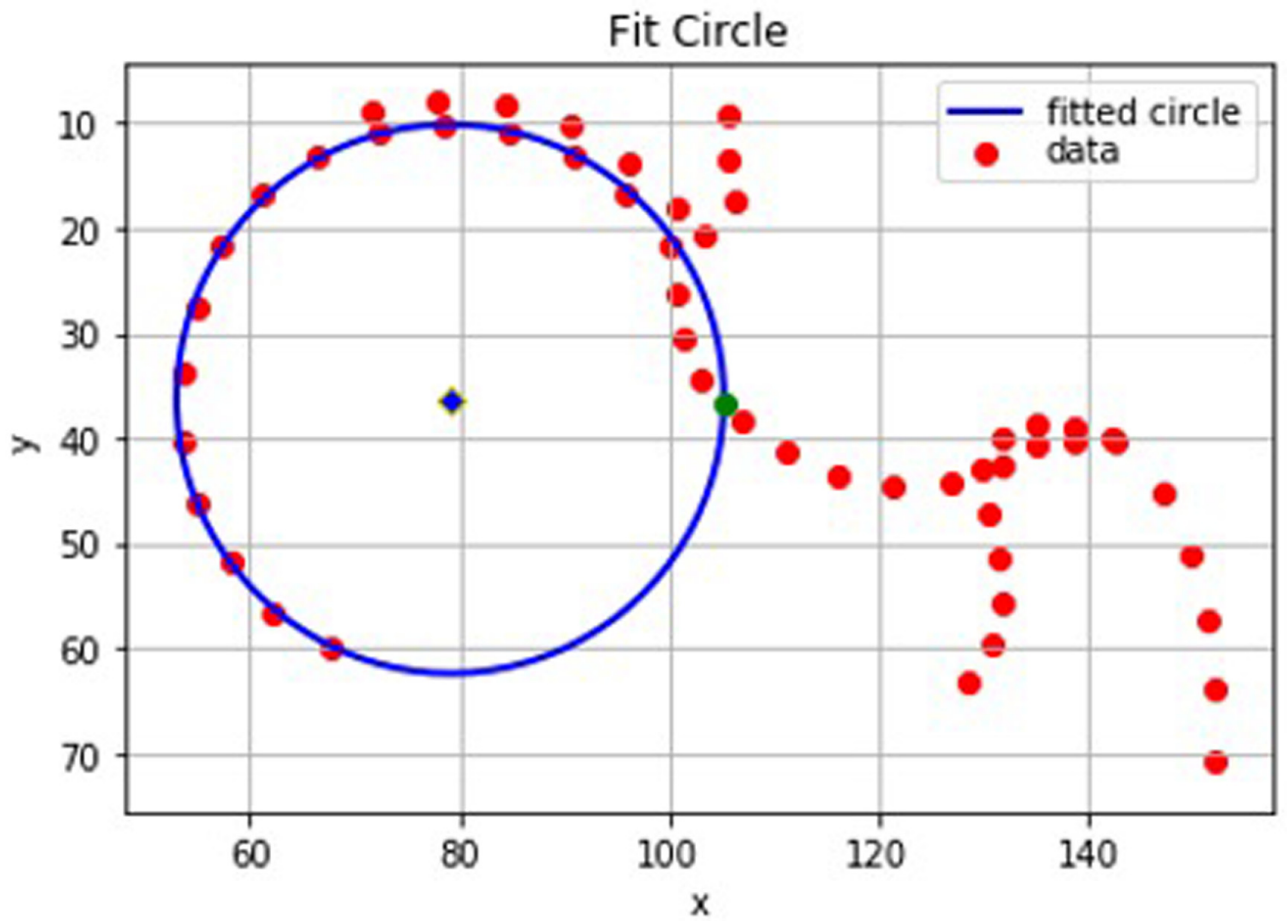
A graphical representation from Python of the points on a ‘straight forward’ dual-energy x-ray absorptiometry (DXA) scan. In this case the intersection position (green) is easily defined between two outline points which lie either side of the circle of best fit.

**Figure 5.**
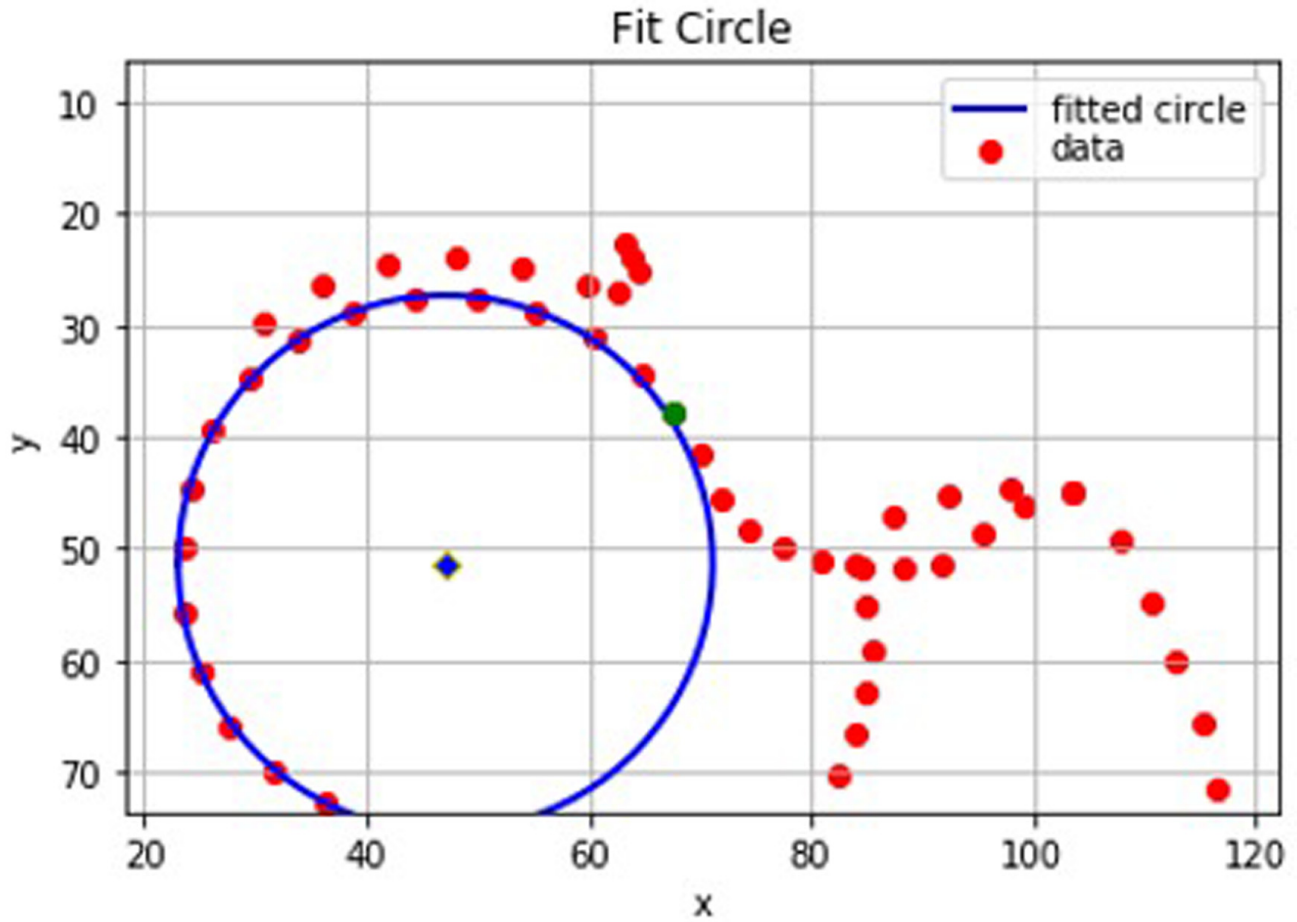
A graphical representation from Python of a set of points where the line between the index point and its preceding point does not intersect the circle. In this case, the preceding point (green) is the approximate intersection position as it has a smaller residual than the index point.

Once the index point has been identified, the intersection position can be calculated. The calculation of the intersection position depends on whether the outline point preceding the index point lies inside or outside of the circle of best fit. In the former case the intersection position is defined by the coordinates at which a line between the index point and the preceding point crosses the circle ( Figure 4). If the outline point preceding the index point lies outside of the circle, but within the index point threshold, then there is no clear intersection position ( Figure 5). In this case, the intersection position is approximated to be the outline point before the index point. This is also the case if the line between the index point and preceding point is a tangent to the circle where again there is no clear intersection position.

### Model 2 – index point definition change

**Figure 6.**
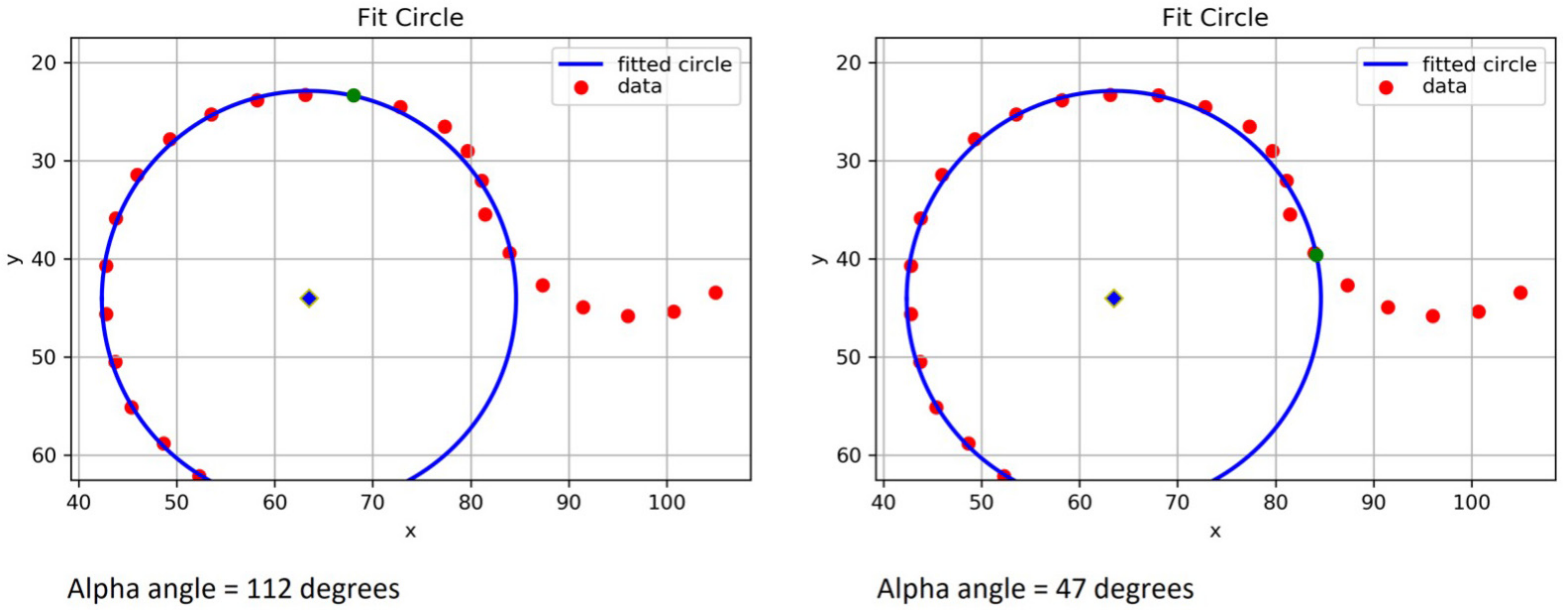
A graphical representation from Python of the same set of points for Experiments 1 and 2. The blue circle shows the circle of best fit plotted from point 15 to 28. The left image shows the results of Experiment 1 with the green interception point marked at around 12 o’clock giving an automatic alpha angle (AA) of 112°. This was viewed as a falsely elevated AA as on visual inspection of this hip dual-energy x-ray absorptiometry (DXA) scan. The slight deviation seen on this graphical illustration of the points from 1 to 2 o’clock was not visible by eye. The right image shows the same set of points for Experiment 2 with the green interception point marked close to 3 o’clock, giving an automatic AA of 47°. The manual AA for this image was 46°. Experiment 2 yielded improved results because of the optimised definition of the index point.

Initial testing showed that the index point definition was too sensitive in *model 1*, identifying outline points as having left the circle which by manual visual inspection appeared to be on the circle. To improve performance and decrease the ‘false positive rate’ of cam detection, the index point definition was changed in *model 2,* to now require three consecutive points leaving the circle by increasing residual values above the index point threshold ( Figure 6). *Model 2* was no different to *model 1* with regards to the other key elements: circle fitting, femoral neck midpoint and intersection position.

Testing the performance of *model 2* against manual measures in 100 randomly selected images showed good agreement but there were only two images with manually classified cam morphology in this sample. This led to the suspicion that the high percentage agreement (see *Results*) achieved in this experiment may be due to the sample being a poor testing set. *Model 2* was then tested again using a weighted random sample such that one third (33%) of the images had an automatic AA ≥60°. This improved the discriminatory performance for cam morphology compared to *model 1. However,* it was still deemed that there were too many ‘false positive’ results where AA was too high as compared to manual assessment. From here on all testing subsamples were weighted to include one third (33%) of images with an automatic AA ≥60°.

### Model 3 – refined index and intersection position definitions

To further improve the index point threshold for *model 3*, negative residuals were included for the first time (after multiplication by -1) and could now inflate the index point threshold; if points 15–28 lay inside the circle then their negative residual might show greater deviation from the circle than those outline points which lay outside of the circle with positive residuals. Moreover, a minimum index point threshold of 1mm was included for cases of near perfect circle fit (i.e. cases where the femoral head was deemed by the automatic method to have left the circle but this was not discernible manually), aiming to reduce ‘false positives’. A value of 1mm was selected as the minimum threshold to represent an approximation of what was detectable by eye on the images. Prior to the minimum index point threshold, a point lying a fraction of a millimetre outside of the circle could be incorrectly registered as the index point (e.g. for very spherical femoral heads). In addition, if there was no intersection position on the circle then the approximated intersection position was moved to the outline point before, on or after the index point depending on which outline point had the smallest residual. Otherwise, *model 3* remained the same as *model 2* in terms of circle fitting and the femoral neck mid-point.

Qualitative analysis of *model 3* results and in particular outliers indicated the index point definition had become too stringent with several ‘false negative’ scans (i.e. non-detected cam morphologies).

### Model 4 – final model

For the fourth model iteration ( *model 4*), the index point definition was modified so that the residual for three consecutive points following the index point had to be greater than the index point threshold but they did not need to be ever increasing. That is, the point after the index point could have a smaller residual than the index point as long as it remained above the index point threshold. Otherwise *model 4* remained the same as *model 3* with regards to circle fitting, femoral neck mid-point and the intersection position.

### Statistical analysis

We report the mean AA along with the AA range for both manual and automatic measures. To assess agreement between manual and automatic AA measures, the mean absolute difference is presented along with its standard deviation (SD) and a concordance correlation coefficient was calculated, presented with its 95% confidence interval (CI) ^26^. Bland-Altman plots were used to visualise this agreement and to identify outliers. Cam morphology was defined as AA ≥60° based on a recent systematic review of previous studies ^8^. To give a broad perspective on the automatic classification of cam, the prevalence of cam morphology, derived from the automatically calculated AA, is given for each model for either the training sample (n=1,930) or the extension sample (n=4,877) depending on the test population. Due to the known AA differences between sexes (i.e. females tend to have a lower AA than males) the summary results of our final model were stratified by sex ^6^. In addition, inter-rater kappa statistics, presented with percentage agreement, were used to compare the cam morphology classifications based on the manual versus automatic AA measurements. Following the described iterative approach, we aimed to achieve strong agreement between the manual and automatic cam classifications as defined by a target kappa of 0.8 ^27^. All statistical analysis was performed using Stata version 15 (StataCorp, College Station, TX, USA).

## Results

A description of basic demographic variables is provided for the training (n=1930), extension (n=4877) and combined samples (n=6807) in Table 1. Each model 1–4 derived AA from the manually placed points in the training sample (n=1930) and these measures were tested against manually derived AA in four subsets of 100 images. Mean manual and automatic AA including ranges, mean absolute difference, concordance correlation coefficients and cam-based inter-rater kappa’s for each model iteration are given in Table 2.

**Table 1.** Sample demographics. This table shows the age, height, weight and sex of the individuals in the training, extension and combined samples. The combined sample is the training and extension sample together.

Demographic variables Continuous	Training sample Mean [range]	Extension sample Mean [range]	Combined sample Mean [range]
Age (years)	62.3 [45-78]	62.3 [46-80]	62.7 [45-80]
Height (cm)	169.8 [145-198]	170.2 [137-203]	170.1 [137-203]
Weight (kg)	76.8 [42-154]	76.0 [36-160]	76.2 [36-160]
Demographic variables Binary	Training sample Count [percentage]	Extension sample Count [percentage]	Combined sample Count [percentage]
Sex (male)	949 [49.2]	2433 [49.9]	3382 [49.7]
Sex (female)	981 [51.8]	2444 [50.1]	3425 [50.3]
Total (n)	1930	4877	6807

**Table 2.** Comparing of the performance of each automatic model iteration versus manual measurement of alpha angle (AA). The manual and automatic means are given for each set of images (n=100) compared in the respective testing samples. The mean absolute difference calculated between the manual and automatic means is presented with its standard deviation (SD). The inter-rater kappa was calculated between manual and automatic cam classifications. The percentage agreement is presented alongside the kappa statistic. The concordance correlation coefficient compares the continuous alpha angle measures and is presented with a 95% confidence interval (CI). The prevalence of cam morphology defined as automatic AA ≥60° is given for all 1930 participants in the training sample for model iteration 1-4 and all 4,877 participants from the extension sample in the final model test.

Model iteration	Test sample	Manual AA Mean [Range]	Automatic AA Mean [Range]	Mean absolute difference [SD]	Kappa [percentage agreement]	Concordance correlation coefficient [95% CI]	Automatic cam prevalence
1	Random training sample	49.5 [36.4-115.8]	60.2 [38.0-117.9]	10.7 [25]	0.43 [80%]	0.28 [0.13-0.43]	28.7%
2	Random training sample	44.1 [34.2-77.3]	46.9 [36.3-103.3]	2.8 [6.9]	0.66 [98%]	0.60 [0.52-0.69]	9.6%
2	Random and weighted training sample	56.9 [36.7-103.7]	66.7 [39.8-113.2]	9.8 [16]	0.59 [80%]	0.62 [0.52-0.72]	9.6%
3	Random and weighted training sample	56.9 [36.7-103.7]	59.1 [39.1-106.2]	2.1 [11.0]	0.70 [87%]	0.81 [0.74-0.87]	4.7%
4	Random and weighted training sample	53.8 [35.3-100.6]	56.0 [35.5-106.2]	2.2 [10.8]	0.84 [93%]	0.83 [0.77-0.89]	7.2%
Final model	Random and weighted extension sample	54.7 [32.4-99.0]	58.1 [33.2-102.0]	3.4 [8.6]	0.86 [94%]	0.88 [0.84-0.92]	9.1%

### Final model – model 4

**Figure 7.**
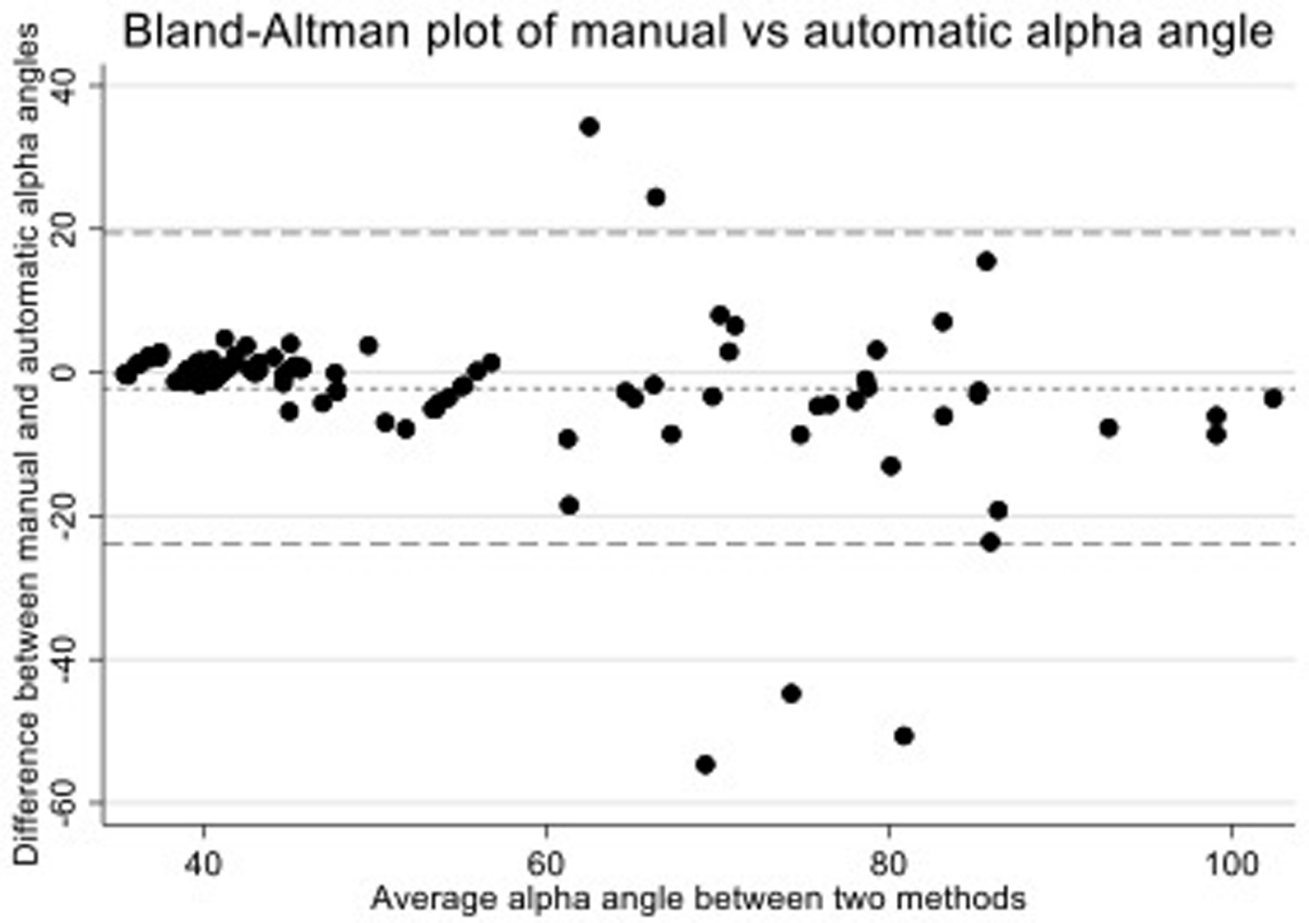
A Bland-Altman plot for *model 4* testing in the training sample to compare manual and automatic alpha angles. The figure shows that all measures were within a 95% confidence interval (dashed lines) apart from 5 outliers. All outliers were examined and visually inspected: four outliers showed errors in the manual AA measure where the automatic measure was able to better fit a circle to the femoral head; and one outlier where the automatic method ignored a slight bulging of the femoral head as one outline point had a residual of 0.96mm, 0.04mm below the threshold of 1mm.

In 100 randomly selected images from the training sample, weighted to include one third (33%) with an automatic AA ≥ 60°, *model 4* gave an automatic mean AA of 56.0° [range 35.5–106.2°] compared to a manual mean AA of 53.8° [35.3-100.6°]. The mean absolute difference was 2.2° [SD 10.8]. The concordance correlation coefficient was 0.83 [95% CI: 0.77-0.89] and the cam-based inter-rater kappa was 0.84 [93% agreement] ( Table 2). A Bland-Altman plot ( Figure 7) showed only five of the 100 images lay outside of the 95% confidence interval; all of the five outliers had higher than average AAs. On review of the five outlier images, four showed errors in the manual AA measurement with poor manual circle fitting. The remaining image showed the automatic method had failed to recognise a visually noticeable deviation of the femoral head from the circle of best fit; the residual for one of the three outline points encompassing this deviation was 0.96mm (0.04mm beneath the automatic minimum index point threshold of 1mm) meaning the algorithm did not measure the AA from this area. The kappa statistic for *model 4* was above the target threshold of 0.8 meaning it was selected as the final model to test in the extension sample.

### Testing the final model in the extension sample

Our final model ( *model 4*) was applied to the extension sample of 4,877 individuals with automatically placed outline points which were subsequently manually corrected. From the extension sample, a randomly selected subsample of 100 DXA scans, weighted to include one third (33%) of images with an automatic AA ≥60°, had manual AA derived. Comparison between automatic AA based on points that were automatically derived but manually corrected and manual AA showed an automatic mean AA of 58.1° [33.2-102.0°] compared to a manual mean AA of 54.7° [32.4-99.0°]. The mean absolute difference was 3.4° [SD 8.6]. The concordance correlation coefficient was 0.88 [95% CI: 0.84-0.92] and the cam-based inter-rater kappa was 0.86 [94% agreement].

### Manual intra-rater comparison

The subsample of 100 images which was used to test *model 4* in the training sample was re-assessed for manually derived AA after 9 months. The two sets of manual AA measures were compared, giving a concordance correlation coefficient of 0.83 [95% CI: 0.76-0.89] and the cam-based intra-rater kappa was 0.80 (91% agreement).

### Automatic alpha angle in the combined sample

Our final model ( *model 4)* was then applied to all the individuals in the combined sample (n=6807), the mean AA was 47.8° [33.2-115.0°], in males the mean AA was 51.6° [35.8-106.2°] and in females the mean AA was 44.2° [35.2-115.0°].

## Discussion

We propose a method to automatically derive AA from AP hip DXA scans. The method is based on outline points and has been validated against manual AA measures. We have described how the method was developed, providing our Python code for the final version of the method for wider use by the research community ^28^.

Similar methods utilising outline points to calculate AA have been reported previously but these studies do not include details on method synthesis, validation, nor open source code to allow for replication. In addition, some do not incorporate automatically placed points and require full manual annotation of the outline points which is time-consuming ^4, 9^ and those methods including automated point placement failed to achieve consistent results as compared with manual annotators ^15^. In contrast, the work presented here allows for replication of our methods and details our comprehensive validation using 500 blinded manual AA measures. In terms of validation, previously reported studies investigating cam morphology defined by AA have reported inter-rater kappas of 0.73 ^4^ and 0.83 ^29^; our method showed an inter-rater kappa of 0.84-0.86 which compares favourably to these studies. In addition, our automatically derived AA from DXA scans in 6,807 UKB participants (males: mean AA 51.6°, range 35.8–106.2°; females: mean AA 44.2°, range 35.2–115.0°) were closely aligned to AA derived manually from radiographs in a large Danish cohort (males: mean 52.6°, range 30–108°; females: mean 45°, range 26–92°) providing further indication that our methods work as expected ^6^. Interestingly, the final automatic model did record, on average, a 3.4° higher AA than manual annotation. This likely reflects the automated methods capturing deviation of the femoral head from the circle of best fit sooner than the manual annotator.

There are limitations to our work. Firstly, although the outline point placement is automated it requires manual checking to make sure it is correct, and osteophytes are excluded from the outline. However, this requires much less time than manually placing outline points and makes it feasible to obtain the large sample sizes required for genome-wide association studies ^30^. Further work is being undertaken to improve automated outline point placement, and to develop a flagging system to highlight images requiring manual inspection where the point placement is suboptimal. Secondly, when validating our automated method, we compared these measures to one highly trained manual operator only. Another limitation is the DXA scan positioning used in this study included a broad range of internal rotation tolerated (15–25°). That said, this probably makes our study more representative of clinical imaging where positioning can vary considerably. In addition, this code was developed using DXAs of only left hips which might be systematically different in shape to right hips. In an independent study, we have applied these methods to both right and left hip AP radiographs (the right hip images were mirrored before applying the outline points and code), validating the results with manual measures from two clinical annotators ^31^. The automated methods performed well against manual measures in this study but more work is needed to assess the reproducibility of these results in external cohorts and clinical environments. A main contribution of this work is that this is the first paper to set out a detailed method of how to automatically derive measures of AA on AP hip images. More work is needed to see if and how the proposed method can be successfully repurposed for lateral hip radiographs.

To conclude, we have described the development and validation of a method to derive AA on AP hip DXA images. We have made the proposed method available to other researchers in the field, allowing for AA to be derived in a standardised way across studies and in particular large population cohorts. This will enable the analysis of AA against clinically relevant outcomes such as OA, hip pain and THR, paving the way for this technology to be integrated into clinical care.

## Data availability

### Source data

The outline points for the UKB hip DXA scans used in this study are developed by the Wellcome Collaborative Grant AUGMENT (project application 17295). The points files used in this study will be made available from UK Biobank and they will be contained in a subsequent data release. UK Biobank control the image specific data developed as part of this research (i.e. points files) and hence they cannot be uploaded to a separate repository. UK Biobank resources are open to all researchers which will allow for replication.

## Software availability

Source code available from: https://github.com/benfaber20/Automatic-alpha-angle/tree/v1.2. Archived source code at time of publication: https://doi.org/10.5281/zenodo.4462770 ^28^.

License: GNU General Public License
