# Supplementary material for: Deriving alpha angle from anterior-posterior dual-energy x-ray absorptiometry scans: an automated and validated approach
Source: Wellcome Open Res. 2022 Jul 19;6:60. Originally published 2021 Mar 15. [Version 2] doi: 10.12688/wellcomeopenres.16656.2 (PMC9426635; doi:10.12688/wellcomeopenres.16656.2)
Supplement: Peer review history

# Peer review: Reviewer response for version 2

**DOI:** 10.21956/wellcomeopenres.20024.r51583

**Published:** 2022-07-29

**Authors:** Pim van Klij

**Affiliations:** 1 Sports Medicine, Isala , Zwolle, The Netherlands

I am happy with the way my comments are addressed. I have no additional comments. Great to be involved in this paper. Happy to be involved to help with other manuscripts in the future.

Is the rationale for developing the new method (or application) clearly explained?

Yes

Is the description of the method technically sound?

Yes

Are the conclusions about the method and its performance adequately supported by the findings presented in the article?

Yes

If any results are presented, are all the source data underlying the results available to ensure full reproducibility?

Partly

Are sufficient details provided to allow replication of the method development and its use by others?

Yes

Reviewer Expertise:

Hip and groin problems in athletes with specific interest for cam morphology development.

I confirm that I have read this submission and believe that I have an appropriate level of expertise to confirm that it is of an acceptable scientific standard.

# Peer review: Reviewer response for version 1

**DOI:** 10.21956/wellcomeopenres.18367.r50806

**Published:** 2022-06-15

**Authors:** James Masters

**Affiliations:** 1 Oxford Trauma, Nuffield Department of Orthopaedics, Rheumatology and Musculoskeletal Sciences, University of Oxford, Oxford, UK

Summary: A technical validation piece of work on automated measurement of the Alpha angle from DEXA scans.

 Strengths: Large numbers of images used, robust processes appropriately comparing to gold standard diagnostic techniques. 

 Well reported with sufficient detail to reproduce work. 

 Considerations: Only left hips are used. There is a school of thought that considers FAI part of a pathoanatomic spectrum with DDH. DDH is recognised to be more prevalent on the left hip due to intrauterine moulding. I therefore wonder whether some additional validation on a right hip data set would be valuable or of interest if available.

 For the manual measure of the alpha angle, there may be value in an additional manual rater, particularly as there is a consistent difference between the manual and automated methods.  

 Overall a well executed piece of work which will add to the field.

Is the rationale for developing the new method (or application) clearly explained?

Yes

Is the description of the method technically sound?

Yes

Are the conclusions about the method and its performance adequately supported by the findings presented in the article?

Yes

If any results are presented, are all the source data underlying the results available to ensure full reproducibility?

Yes

Are sufficient details provided to allow replication of the method development and its use by others?

Yes

Reviewer Expertise:

Clinical effectiveness research with particular interest in randomised trials in trauma and orthopaedic surgery

I confirm that I have read this submission and believe that I have an appropriate level of expertise to confirm that it is of an acceptable scientific standard.

# Response

**Published:** 2022-07-13

**Authors:** Ben Faber

**Affiliations:** The Musculoskeletal Research Unit, University of Bristol & North Bristol NHS Trust, UK

*Summary: A technical validation piece of work on automated measurement of the Alpha angle from DEXA scans.*

*Strengths: Large numbers of images used, robust processes appropriately comparing to gold standard diagnostic techniques.*

*Well reported with sufficient detail to reproduce work.*

*Considerations: Only left hips are used. There is a school of thought that considers FAI part of a pathoanatomic spectrum with DDH. DDH is recognised to be more prevalent on the left hip due to intrauterine moulding. I therefore wonder whether some additional validation on a right hip data set would be valuable or of interest if available.*

 Author response:

 We now mention this point in our limitations. “In addition, this code was developed using DXAs of only left hips which might be systematically different in shape to right hips.” Since submitting this paper to *WOR* we have applied this method to a cohort of left and right AP hip radiographs and published the results. We are now referring to the latter in the paper as follows: “In an independent study, we have applied these methods to both right and left hip AP radiographs (the right hip images were mirrored before applying the outline points and code), validating the results with manual measures from two clinical annotators ^31^.” In this study we examined left and right hips with no discernible side-based systematic errors encountered when comparing our automated readings with manual annotation.

*For the manual measure of the alpha angle, there may be value in an additional manual rater, particularly as there is a consistent difference between the manual and automated methods.*

 Author response:

 We acknowledge this limitation and mention it as such. In addition, we now include a reference to a study where this code was applied to radiographs and validated against two manual annotators. This now reads “In an independent study, we have applied these methods to both right and left hip AP radiographs (the right hip images were mirrored before applying the outline points and code), validating the results with manual measures from two clinical annotators ^31^.”

# Peer review: Reviewer response for version 1

**DOI:** 10.21956/wellcomeopenres.18367.r48970

**Published:** 2022-04-07

**Authors:** Pim van Klij

**Affiliations:** 1 Sports Medicine, Isala , Zwolle, The Netherlands

Thank you for the invitation to review this manuscript. It is a well-organized manuscript with some new information regarding the most used measurement for cam morphology. It might introduce some new insights in how we can automize these measurements in bigger cohorts. Despite being quite familiar with hip/groin problems and the alpha angle measurement, the extensive described technique behind it is still a bit outside my expertise.

**Title: **no suggestions.

**Abstract:**

- Introduction: would suggest to write down ‘imaging measure’ to make sure that the reader understands it is about an imaging measurement.

**Introduction**

 Alinea 1:

- A recent systematic review (van Klij et al. 2020) tried to clear up the mess around the alpha angle threshold for cam morphology, might be good to refer to this paper early in the manuscript. ^1^

Alinea 2:

- In FAI syndrome, it is not only pain/symptoms, but also clinical signs that got involved, please introduce this here as well.
- Other software used for AA measurement can be MATLAB, might be good to add this here as it is also widely used.

*Figure 1*: I would suggest to not use capitals for ‘Morphology’ and ‘Angle'. And maybe the quality of the circle and lines could be a bit better, as lines are crossing the red circle or even gaps between.

**Methods**

 General: I believe the Methods section is your most important section, but however due to the very extensive explanation of the process, it makes it hard to read for a reader who is not that familiar with these measures. You might think of reducing it a bit to keep the reader onboard.

*Population*

- Is it possible to get more insight in what questionnaire is used to define the OA diagnosis?
- I do not find any information about informed consent for these participants and by which University this study is approved. Please introduce some medical ethical insight for the reader.

*DXA images and outline point*

- Supine position I suppose? Please add.
- Why the 15 to 25 degrees difference/margin? And why only the left hips, this is simply loss of power? For analysis, you could simply mirror right side images, right?

*Figure 2:* My suggestion would be to increase font size for the numbers in the figure to help guide the reader through the process with all numbered dots.

*Statistical analysis*

- By ‘the known AA differences between sexes’, please explain briefly for the more unexperienced reader that you mean that AA in general are lower in females.

**Results**

- Please introduced the numbers (n) also in Table 1 to make it more readable.

*Final model – model 4*

- Was the difference between manually and automatic measurement statistically significant? It might be interesting for the reader to give more insight in this and to objectively decide what is best in their opinion.

*Testing the final model in extension sample*

- It seems that the automatic model measures higher AA’s that the manually placed ones, it seems that the outliers outside the 95% confidence interval are investigated further but how about outliers that are close to that range?

**Discussion**

- I still do not read an explanation for the differences found in general between automatic and manually placed dots for AA measurement. In general higher AAs were found in the automatic group, how can this be explained? What is most reliable and why? What can explain this difference?
- How can this measurement method be used for lateral radiographs? Is it one-on-one reproducible?
- Can this method also be used by a more unexperienced clinician in the field? If not, why not? We need to work towards a system which fits for (nearly) all clinicians who want to perform a reliable measurement.
- Just a question, do you believe this method can be used for the younger audience? Did not find information about this. As younger athletes mostly develop cam morphology in their adolescence with an open growth plate, would this measurement method system be useful for all ages?

In general, great manuscript with surely additional information for the reader. Thank you for inviting me to review it. Looking forward to correction of the manuscript.

Is the rationale for developing the new method (or application) clearly explained?

Yes

Is the description of the method technically sound?

Yes

Are the conclusions about the method and its performance adequately supported by the findings presented in the article?

Yes

If any results are presented, are all the source data underlying the results available to ensure full reproducibility?

Partly

Are sufficient details provided to allow replication of the method development and its use by others?

Yes

Reviewer Expertise:

Hip and groin problems in athletes with specific interest for cam morphology development.

I confirm that I have read this submission and believe that I have an appropriate level of expertise to confirm that it is of an acceptable scientific standard, however I have significant reservations, as outlined above.

## References

[1] : Classifying Cam Morphology by the Alpha Angle: A Systematic Review on Threshold Values. *Orthop J Sports Med* .2020;8(8) : 10.1177/2325967120938312 2325967120938312 10.1177/2325967120938312 32844100PMC7418265

# Response

**Published:** 2022-07-13

**Authors:** Ben Faber

**Affiliations:** The Musculoskeletal Research Unit, University of Bristol & North Bristol NHS Trust, UK

We thank the reviewers for their feedback and constructive comments.

*Reviewer 1:*

*Thank you for the invitation to review this manuscript. It is a well-organized manuscript with some new information regarding the most used measurement for cam morphology. It might introduce some new insights in how we can automize these measurements in bigger cohorts. Despite being quite familiar with hip/groin problems and the alpha angle measurement, the extensive described technique behind it is still a bit outside my expertise.*

***Title: **no suggestions.*

***Abstract:***

- *Introduction: would suggest to write down ‘imaging measure’ to make sure that the reader understands it is about an imaging measurement.*

Author response:

 We have made this change.

***Introduction***

  

- *A recent systematic review (van Klij et al. 2020) tried to clear up the mess around the alpha angle threshold for cam morphology, might be good to refer to this paper early in the manuscript.* *^1^*

Author response:

 We have now written the following which includes the reference you suggest “The higher the AA the more indicative of cam morphology it is – with previously published thresholds of 50°, 55°, 60° and 83° all being used to define the presence of cam morphology ^1, 4– 7^, and with a recent systematic review concluding a threshold of 60° is best ^8^.”

  

- *In FAI syndrome, it is not only pain/symptoms, but also clinical signs that got involved, please introduce this here as well.*

Author response:

 We have amended that sentence to now read “Cam morphology is thought to lead to pain and limited function of the hip in FAI syndrome.”

- *Other software used for AA measurement can be MATLAB, might be good to add this here as it is also widely used.*

Author response:

 We now clarify that MATLAB is used in the references we give. “Alternatively, AA has been calculated using MATLAB based software which utilises outline points that have been placed manually around the femoral head.”

- *Figure 1: I would suggest to not use capitals for ‘Morphology’ and ‘Angle'. And maybe the quality of the circle and lines could be a bit better, as lines are crossing the red circle or even gaps between.*

Author response:

 We have removed the text from the image as it is in the legend. In addition, all the lines now intersect the circle of best fit.

***Methods***

- *General: I believe the Methods section is your most important section, but however due to the very extensive explanation of the process, it makes it hard to read for a reader who is not that familiar with these measures. You might think of reducing it a bit to keep the reader onboard.*

Author response:

 We have tried to keep things as brief as possible. We believe it is necessary to include sufficient detail for the reader to be able to assess the resultant code.  

*Population*

- *Is it possible to get more insight in what questionnaire is used to define the OA diagnosis?*

Author response:

 We have now included more information on the questionnaire used to define the OA diagnosis. The text now reads “The first 20% of both the training and extension samples were selected randomly from those with a self-reported diagnosis of OA based on a questionnaire completed at the same visit as the DXA scan. In the questionnaire, each participant was asked to record all their known diagnoses and a trained nurse subsequently coded the diagnosis. The OA code was not site specific.”

- *I do not find any information about informed consent for these participants and by which University this study is approved. Please introduce some medical ethical insight for the reader.*

Author response:

 We have now included this information. The text reads “This study was approved by UKB (application number 17295) which is overseen by its own Ethics Advisory Committee. In addition, UKB received approval from the National Information Governance Board for Health and Social Care and Northwest Multi-Centre Research Ethics Committee (11/NW/0382). All participants provided informed consent.”

*DXA images and outline point*

- *Supine position I suppose? Please add.*

Author response:

 We have now added this.

  

- *Why the 15 to 25 degrees difference/margin? And why only the left hips, this is simply loss of power? For analysis, you could simply mirror right side images, right?*

Author response:

 Thank you. The agreed UKB DXA standard operating procedure dictated the positioning of the hips. This was out of the control of the authors. Aside from this, left hip images were chosen as part of a wider research programme to automatically assess hip shape in UKB with a view to then doing the same on the right hips. As yet only the left hips have been examined due to time constraints but the right hips will be examined in the future. In an independent study, we have now used this code on left and right *radiographs* with good repeatability.

 We now mention both of these issues in the limitations “Another limitation is the DXA scan positioning used in this study included a broad range of internal rotation tolerated (15-25°). That said, this probably makes our study more representative of clinical imaging where positioning can vary considerably. In addition, this code was developed using DXAs of only left hips which might be systematically different in shape to right hips. In an independent study, we have applied these methods to both right and left hip AP radiographs (the right hip images were mirrored before applying the outline points and code), validating the results with manual measures from two clinical annotators ^31^.”

- *Figure 2: My suggestion would be to increase font size for the numbers in the figure to help guide the reader through the process with all numbered dots.*

Author response:

 We have now increased the font size.

*Statistical analysis*

- *By ‘the known AA differences between sexes’, please explain briefly for the more unexperienced reader that you mean that AA in general are lower in females.*

Author response:

 We have now included the following “Due to the known AA differences between sexes (i.e. females tend to have a lower AA than males) the summary results of our final model were stratified by sex"

***Results***

- *Please introduced the numbers (n) also in Table 1 to make it more readable*

Author response:

 The sample numbers are already included in Table 1 in the last row. We have now modified the description of the row and columns to express this more clearly. The totals in each sample are listed in the text “A description of basic demographic variables is provided for the training (n=1930), extension (n=4877) and combined samples (n=6807) in Table 1.”

*Final model – model 4*

- *Was the difference between manually and automatic measurement statistically significant? It might be interesting for the reader to give more insight in this and to objectively decide what is best in their opinion.*

Author response:

 We did not look at p-values nor p-value thresholds for “statistical significance” for the difference between the manual and automatic AA values as it is more appropriate to assess two types of measurements looking at agreement. The kappa values, concordance correlation coefficients and absolute mean differences provide an objective view of the data and allow assessment of the agreement of the two methods.

*Testing the final model in extension sample*

- *It seems that the automatic model measures higher AA’s that the manually placed ones, it seems that the outliers outside the 95% confidence interval are investigated further but how about outliers that are close to that range?*

Author response:

 These images were inspected and generally it is hard to see much difference between where the femoral head is adjudged to have left the circle. A small difference in distance (e.g. <1mm) between where annotators (e.g. manual or automated) think the femoral head leaves the circle can result in an AA that is several degrees higher. The automatic method recording a higher AA than the manual annotator suggests it declares the femoral head leaving the circle systematically earlier than the manual annotator. This is now mentioned in the discussion “Interestingly, the final automatic model did record, on average, a 3.4° higher AA than manual annotation. This likely reflects the automated methods capturing deviation of the femoral head from the circle of best fit sooner than the manual annotator.”

***Discussion***

- *I still do not read an explanation for the differences found in general between automatic and manually placed dots for AA measurement. In general higher AAs were found in the automatic group, how can this be explained? What is most reliable and why? What can explain this difference?*

Author response:

 Please see the response to the previous point. In terms of reliability, more work is needed to assess these methods in a clinical environment to assess reliability. However, given the automatic measures are more objective one would expect them to be more reliable. We have added the following limitation “The automated methods performed well against manual measures in this study but more work is needed to assess the reproducibility of these results in external cohorts and clinical environments.”

- *How can this measurement method be used for lateral radiographs? Is it one-on-one reproducible?*

Author response:

 These methods should be applicable to lateral radiographs but we have not validated this and it is beyond the scope of this study. We have included this point in the following sentence “More work is needed to see if and how the proposed method can be successfully repurposed for lateral hip radiographs.”

- *Can this method also be used by a more unexperienced clinician in the field? If not, why not? We need to work towards a system which fits for (nearly) all clinicians who want to perform a reliable measurement.*

Author response:

 Currently, the method has been developed as a research tool and has not been used in a clinical environment. The motivation for publishing this paper and code is for others to try. We would welcome collaborations to apply these methods clinically and we have plans to do so ourselves in future.

- *Just a question, do you believe this method can be used for the younger audience? Did not find information about this. As younger athletes mostly develop cam morphology in their adolescence with an open growth plate, would this measurement method system be useful for all ages?*

Author response:

 We believe this method will work as long as the outline points are placed robustly. Our semi-automated approach facilitates this. For severely deformed hips some manual correction of points may be required. Young adults are unlikely to have joint space narrowing, osteophytes, enthesophytes etc, features which make it more difficult to automatically place the outline points. Thus, we would expect the methods to perform equally well or better in young adults. We would welcome collaborations to apply this method in other populations (although with the code being available the method could be used without our direct involvement).
